# Supplementary material for: Wss1 metalloprotease partners with Cdc48/Doa1 in processing genotoxic SUMO conjugates
Source: eLife. 2015 Sep 8;4:e06763. doi: 10.7554/eLife.06763 (PMC4559962; doi:10.7554/eLife.06763)
Supplement: Supplementary file 1. — Wss1-interacting proteins identified by mass spectrometry. DOI: http://dx.doi.org/10.7554/eLife.06763.034 [file elife06763s005.docx]

**Supplementary File 1. Wss1-interacting proteins identified by mass spectrometry.**

| **Name** | **Reference** | **Accession No** | **M.W.** | **pI** | **ProteIn Score** | **ProteIn Score C.I. %** | **Peptide Count** | **Total Ion Score** |
| --- | --- | --- | --- | --- | --- | --- | --- | --- |
| **wss1** | **YHR134W** | **gi\|6321926** | **30607.7** | **8.85** | **159** | **100** | **7** | **134** |
| **doa1** | **YKL213C** | **gi\|6322636** | **79456.1** | **4.87** | **143** | **100** | **8** | **122** |
| **cdc48** | **YDL126C** | **gi\|6320077** | **91955** | **4.66** | **90** | **100** | **11** | **35** |
| tph3 | YJL016W | gi\|1006733 | 19729.5 | 5.94 | 50 | 99.246 | 8 | 9 |
| sso1 | YPL232W | gi\|11514685 | 22752.5 | 5.3 | 47 | 98.628 | 8 | 2 |
| dia2 | YOR080W | gi\|151945706 | 86770.3 | 9.22 | 44 | 97.263 | 12 |  |
| dgr2 | YKL121W | gi\|6322728 | 95961.4 | 8.64 | 41 | 94.012 | 12 | 8 |
| csf1 | YLR087C | gi\|151941254 | 337874.8 | 5.64 | 39 | 90.509 | 22 |  |
| imh1 | YLR309C | gi\|6323340 | 105161.6 | 5.52 | 39 | 89.593 | 17 |  |
| prp45 | YAL032C | gi\|5241 | 44347.3 | 10.17 | 38 | 88.323 | 11 |  |
| hem13 | YDR044W | gi\|6320249 | 37687.8 | 6.32 | 38 | 88.323 | 7 | 6 |
| pxl1 | YKR090W | gi\|6322943 | 79398.2 | 5.08 | 37 | 86.281 | 11 |  |
| pep3 | YLR148W | gi\|6323177 | 107330.1 | 6.51 | 37 | 83.506 | 13 |  |
| sen1 | YLR430W | gi\|172574 | 239201.1 | 8.86 | 36 | 81.494 | 21 |  |
| ppm2 | YOL141W | gi\|6325049 | 89748.2 | 8.48 | 35 | 75.604 | 11 | 5 |
| rat1 | YOR048C | gi\|151945677 | 115861.1 | 6.37 | 34 | 72.628 | 12 |  |
| trm3 | YDL112W | gi\|6320091 | 164943.3 | 5.74 | 34 | 72.628 | 15 |  |
| prr1 | YKL116C | gi\|151941688 | 58929 | 5.78 | 34 | 70.67 | 10 |  |
| ret1 | YOR207C | gi\|6324781 | 129373.6 | 8.4 | 33 | 61.335 | 14 |  |
| msn5 | YDR335W | gi\|151942311 | 131870.5 | 5.12 | 33 | 63.916 | 13 |  |
| clb1 | YGR108W | gi\|151943389 | 54863.7 | 5.65 | 32 | 54.573 | 8 | 6 |
| ubp6 | YFR010W | gi\|14318532 | 57075 | 6.71 | 32 | 52.432 | 7 | 7 |
| myo1 | YHR023W | gi\|6321812 | 223498.3 | 6.06 | 32 | 52.432 | 15 | 23 |
| YDR306C | YDR306C | gi\|6320512 | 54401.9 | 8.8 | 32 | 51.324 | 8 |  |
| chl4 | YDR254W | gi\|151942230 | 52624.4 | 8.8 | 31 | 38.721 | 8 |  |
| thp3 | YPR045C | gi\|151942832 | 53699.1 | 9.1 | 31 | 37.293 | 7 |  |
| rad2 | YGR258C | gi\|51013165 | 117731 | 5.08 | 31 | 41.479 | 9 | 12 |
| cpr3 | YML078W | gi\|6323562 | 19906.1 | 8.81 | 30 | 19.218 | 5 |  |
| uso1 | YDL058W | gi\|4778 | 206299.8 | 4.88 | 30 | 29.642 | 17 |  |
| tra1 | YHR099W | gi\|6321891 | 432905 | 6.11 | 30 | 19.218 | 24 | 5 |
| arp3 | YJR065C | gi\|6322525 | 49510.9 | 5.57 | 29 | 0.617 | 7 |  |
| rsc4 | YKR008W | gi\|51013697 | 72245.4 | 5.15 | 29 | 11.424 | 9 |  |
| rpl17 | YKL180W | gi\|6322668 | 20537.1 | 10.92 | 29 | 13.441 | 6 |  |
| nvj2 | YPR091C | gi\|151942870 | 87142.7 | 6.95 | 29 | 9.361 | 9 |  |
| fir1 | YER032W | gi\|603624 | 104578.4 | 7.42 | 29 | 7.25 | 11 |  |
| rad10 | YML095C | gi\|6323543 | 24296.3 | 8.87 | 29 | 0.617 | 2 | 21 |
| msh6 | YDR097C | gi\|1588283 | 140075.1 | 5.56 | 29 | 0 | 1 |  |
| rrm3 | YHR031C | gi\|6321820 | 81530.2 | 9.69 | 28 | 0 | 10 |  |
| pac2 | YER007W | gi\|151944714 | 59172.5 | 8.68 | 28 | 0 | 8 |  |
| dcc1 | YCL016C | gi\|10383774 | 44045.8 | 6.04 | 28 | 0 | 7 |  |
| emi2 | YDR516C | gi\|151942477 | 55886.6 | 5.79 | 28 | 0 | 7 |  |
| scl1 | YGL011C | gi\|6321427 | 27983.2 | 5.89 | 28 | 0 | 6 |  |
| ure2 | YNL229C | gi\|22135411 | 40218.8 | 6.01 | 28 | 0 | 6 |  |
| sec72 | YLR292C | gi\|460049 | 21625.3 | 5.56 | 28 | 0 | 5 |  |
| env7 | YPL236C | gi\|2326841 | 6083.1 | 9.06 | 28 | 0 | 3 |  |
| YCR043C | YCR043C | gi\|6319891 | 14769.1 | 5.2 | 28 | 0 | 2 | 18 |
| scc2 | YDR180W | gi\|151942163 | 171040.8 | 6.45 | 27 | 0 | 14 |  |
| pol32 | YJR043C | gi\|6322503 | 40284.5 | 8.5 | 27 | 0 | 6 |  |
| pho88 | YBR106W | gi\|6319582 | 21122.8 | 9.21 | 27 | 0 | 6 |  |
| pap1 | YKR002W | gi\|6322854 | 64511.5 | 7.95 | 26 | 0 | 8 |  |
| rsa4 | YCR072C | gi\|10383804 | 56989.8 | 9.06 | 26 | 0 | 7 |  |
| rpc34 | YNR003C | gi\|151944532 | 36126.3 | 5.27 | 26 | 0 | 6 |  |
| smc3 | YJL074C | gi\|151945253 | 141221.6 | 5.63 | 26 | 0 | 5 | 18 |
| rpn14 | YGL004C | gi\|6321434 | 46354.1 | 5.06 | 25 | 0 | 7 |  |
| erd1 | YDR414C | gi\|6320622 | 43188.1 | 9.38 | 24 | 0 | 6 |  |
