# Supplementary material for: Wss1 metalloprotease partners with Cdc48/Doa1 in processing genotoxic SUMO conjugates
Source: eLife. 2015 Sep 8;4:e06763. doi: 10.7554/eLife.06763 (PMC4559962; doi:10.7554/eLife.06763)
Supplement: Supplementary file 3. — Yeast strains used in this study. DOI: http://dx.doi.org/10.7554/eLife.06763.036 [file elife06763s007.docx]

**Supplementary File 3. Yeast strains used in this study.**

| **NAME** | ***GENOTYPE*** | **REFERENCE** |
| --- | --- | --- |
| BY4742 | *MATα his3Δ1 leu2Δ0 lys2Δ0 ura3Δ0* | Open Biosystem |
| MBY15 | *wss1Δ::KAN* | This study |
| MBY31 | *tdp1Δ::HPH* | This study |
| MBY37 | *wss1Δ::KAN tdp1Δ::HPH* |  |
| MBY17 | *WSS1-MYC13::KAN* | This study |
| MBY18 | *DOA1-FLAG::HIS3* | This study |
| MBY24 | *WSS1-GFP::URA3* | This study |
| MBY34 | *WSS1-GFP::URA3 tdp1Δ::HPH* | This study |
| MBY42 | *pGAL1-MBP-TOP1:: KAN* | This study |
| MBY43 | *pGAL1-MBP-TOP1:: KAN wss1Δ::URA3* | This study |
| MBY44 | *pGAL1-MBP-TOP1:: KAN tdp1Δ::HPH* | This study |
| MBY49 | *pGAL1-MBP-TOP1:: KAN wss1Δ::URA3 tdp1Δ::HPH* | This study |
| MBY46 | *pGAL1-MBP-TOP1:: KAN WSS1-GFP::URA3* | This study |
| MBY47 | *pGAL1-MBP-TOP1:: KAN WSS1-GFP::URA3 tdp1Δ::HPH* | This study |
|  |  |  |
| S288C | *MATa his3Δ1 leu2Δ0 met15Δ0 ura3Δ0* | Open Biosystem |
| Doa1-TAP | *DOA1-TAP::HIS3* | Open Biosystem |
| Cdc48-TAP | *CDC48-TAP::HIS3* | Open Biosystem |
| Top1-TAP | *TOP1-TAP::HIS3* | Open Biosystem |
| MBY19 | *CDC48-TAP::HIS3 wss1Δ::KAN* | This study |
| MBY32 | *CDC48-TAP::HIS3 tdp1Δ::HPH* | This study |
| MBY35 | *TOP1-TAP::HIS3 wss1Δ::KAN* | This study |
| MBY36 | *TOP1-TAP::HIS3 tdp1Δ::HPH* | This study |
| MBY39 | *TOP1-TAP::HIS3 wss1Δ::KAN tdp1Δ::HPH* | This study |
|  |  |  |
| SBY214 | *MATa ura3-1 leu2,3-112 his3-11:pCUP1-GFP12-lacI12:HIS3 trp1-1:lacO:TRP1 ade2-1 can1-100 bar1Δ lys2Δ* | ([Biggins et al., 2001](#_ENREF_1)) |
| MBY14 | SBY214 *wss1Δ::KAN* | This study |
| SBY331 | *MATa ura3-1 leu2,3-112 his3-11:pCUP1-GFP12-lacI12:HIS3 trp1-1:lacO:TRP1 ade2-1 can1-100 bar1Δ lys2Δ smt3-331* | ([Biggins et al., 2001](#_ENREF_1)) |
| MBY13 | SBY331 *wss1Δ::KAN* | This study |
|  |  |  |
| EJY251-11b | *MATα trp1-1 ura3-52 his3-Δ200 leu2-3,112 lys2-801 smt3Δ::HIS3* *p315-PGAL-HFSMT3* | ([Johnson et al., 1997](#_ENREF_2)) |
| MBY21 | EJY251-11b *wss1Δ::KAN* | This study |
| T79 | *MATa ade2-1 can1-100 his3-11,15 leu2-3,112 trp1-1 ura3-1 mms21-11::LEU2* | ([Zhao and Blobel, 2005](#_ENREF_8)) |
