# Supplementary material for: Wss1 metalloprotease partners with Cdc48/Doa1 in processing genotoxic SUMO conjugates
Source: eLife. 2015 Sep 8;4:e06763. doi: 10.7554/eLife.06763 (PMC4559962; doi:10.7554/eLife.06763)
Supplement: Supplementary file 4. — Plasmids used in this study. DOI: http://dx.doi.org/10.7554/eLife.06763.037 [file elife06763s008.docx]

**Supplementary File 4. Plasmids used in this study.**

| **NAME** | ***GENOTYPE*** | **SELECTION** | **REFERENCE** |
| --- | --- | --- | --- |
| **pRSET-B** |  |  |  |
| HA-Wss1-WT | *HA-WSS1* | amp | This study |
| HA-Wss1-WLM* | *HA-WSS1: HE115/116NK* | amp | This study |
| HA-Wss1-SIM1* | *HA-WSS1: VV247/248AA* | amp | This study |
| HA-Wss1-ΔSIM2 | *HA-WSS1(1-259)* | amp | This study |
| HA-Wss1-SIM1*-ΔSIM2 | *HA-WSS1(1-259): VV247/248AA* | amp | This study |
| HA-Wss1-SIM1*-ΔSIM2-WLM* | *HA-WSS1(1-259): VV247/248AA HE115/116NK* | amp | This study |
| HA-N-Wss1 | *HA-WSS1(1-188)* | amp | This study |
| HA-C-Wss1 | *HA-WSS1(188-269)* | amp | This study |
| HA-Wss1-SHP | *HA-WSS1(1-161)* | amp | This study |
| HF-Doa1 | *HF-DOA1* | amp | ([Mullally et al., 2006](#_ENREF_4)) |
| HF-C-Doa1 | *HF-DOA1 (296-715)* | amp | ([Mullally et al., 2006](#_ENREF_4)) |
| HF-WD40 | *HF-DOA1 (1-282)* | amp | ([Mullally et al., 2006](#_ENREF_4)) |
| His6-Cdc48 | *HIS6-CDC48* | amp | This study |
| His6-Ufd1 | *HIS6-UFD1* | amp | This study |
| His6-Npl4 | *HIS6-NPL4* | amp | This study |
| His6-Shp1 | *HIS6-SHP1* | amp | This study |
| Wss1-his6 | *WSS1-HIS6* | amp | This study |
| Wss1-tev-his6 | *WSS1-TEV-HIS6* | amp | This study |
|  |  |  |  |
| **pMAL-c2** |  |  |  |
| MBP-Wss1-WT | *MBP-WSS1* | amp | This study |
| MBP-Wss1-WLM* | *MBP-WSS1: HE115/116NK* | amp | This study |
| MBP-Wss1-SIM1* | *MBP-WSS1: VV247/248AA* | amp | This study |
| MBP-Wss1-ΔSIM2 | *MBP-WSS1(1-259)* | amp | This study |
| MBP-Wss1-R | *MBP-WSS1: R209S* | amp | This study |
| MBP-Wss1-2R | *MBP-WSS1: RR218/219SS* | amp | This study |
| MBP-Wss1-F | *MBP-WSS1: F152S* | amp | This study |
| MBP-Wss1-F2R | *MBP-WSS1: RR218/219SS F152S* | amp | This study |
|  |  |  |  |
| **pGEX4-T1** |  |  |  |
| GST-Cdc48 | *GST-CDC48* | amp | ([Rumpf and Jentsch, 2006](#_ENREF_5)) |
| GST-Cdc48 N | *GST-CDC48 -Ndomain* | amp | ([Rumpf and Jentsch, 2006](#_ENREF_5)) |
| GST-Cdc48 D1D2 | *GST-CDC48 –D1D2domain* | amp | ([Rumpf and Jentsch, 2006](#_ENREF_5)) |
|  |  |  |  |
| **pYEPGAP-URA3** |  |  |  |
| HA-Wss1-WT | *HA-WSS1* | URA3, amp | This study |
| HA-Wss1-WLM* | *HA-WSS1: HE115/116NK* | URA3, amp | This study |
| HA-Wss1-SIM1* | *HA-WSS1: VV247/248AA* | URA3, amp | This study |
| HA-Wss1-ΔSIM2 | *HA-WSS1(1-259)* | URA3, amp | This study |
| HA-Wss1-SIM1*-ΔSIM2 | *HA-WSS1(1-259): VV247/248AA* | URA3, amp | This study |
| HA-Wss1-SIM1*-ΔSIM2-WLM* | *HA-WSS1(1-259): VV247/248AA HE115/116NK* | URA3, amp | This study |
| HA-N-Wss1 | *HA-WSS1(1-203)* | URA3, amp | This study |
| HA-C-Wss1 | *HA-WSS1(204-269)* | URA3, amp | This study |
| HA-Wss1-R | *HA-WSS1: R209S* | URA3, amp | This study |
| HA-Wss1-2R | *HA-WSS1: RR218/219SS* | URA3, amp | This study |
| HA-Wss1-F | *HA-WSS1: F152S* | URA3, amp | This study |
| HA-Wss1-F2R | *HA-WSS1: RR218/219SS F152S* | URA3, amp | This study |
|  |  |  |  |
| **pYEPGAP-TRP1** |  |  |  |
| HA-Wss1-WT | *HA-WSS1* | TRP1, amp | This study |
| HA-Wss1-WLM* | *HA-WSS1: HE115/116NK* | TRP1, amp | This study |
| HA-Wss1-SIM1* | *HA-WSS1: VV247/248AA* | TRP1, amp | This study |
| HA-Wss1-ΔSIM2 | *HA-WSS1(1-259)* | TRP1, amp | This study |
|  |  |  |  |
| **pUG35-URA3** |  |  |  |
| Wss1-GFP | *WSS1-GFP* | URA3, amp | ([Van Heusden and Steensma, 2008](#_ENREF_6)) |
| **pUG36-URA3** |  |  |  |
| GFP-Wss1 | *GFP-WSS1* | URA3, amp | ([Van Heusden and Steensma, 2008](#_ENREF_6)) |
| Wss1-GFP-ΔSIM2 | *GFP-WSS1(1-259)* |  | This study |
|  |  |  |  |
| **pTYB2** |  |  |  |
| SUMO97 | *SMT3(1-97)* | amp | This study |
| Ub75 | *UB(1-75)* | amp | ([Wilkinson et al., 2005](#_ENREF_7)) |
|  |  |  |  |
| His6-Ub-Smt3-HA | *HIS6-UB-SMT3-HA* | amp | ([Li and Hochstrasser, 1999](#_ENREF_3)) |
